# Lymphomas With Primary Gastrointestinal Presentation: A Retrospective Study Covering a Five-Year Period at a Quaternary Care Center in Southern India

**DOI:** 10.7759/cureus.75161

**Published:** 2024-12-05

**Authors:** Dona Maria George, Archana Lakshmanan

**Affiliations:** 1 Department of Histopathology and Cytology, Apollo Main Hospitals, Chennai, IND; 2 Department of Histopathology and Cytology, Apollo Cancer Center, Chennai, IND

**Keywords:** b cell lymphoma, extranodal lymphoma, gastrointestinal lymphoma, hodgkin lymphoma, lymphomas with primary gastrointestinal presentation, non-hodgkin lymphoma, t cell lymphoma

## Abstract

Background and objective

Lymphomas can involve the gastrointestinal (GI) tract as a primary disease or as a secondary spread of systemic disease. The GI tract is a key site for extranodal lymphomas, with Hodgkin lymphoma (HL) and non-Hodgkin lymphoma (NHL) occurring in this region. This study aimed to analyze the demography, anatomic distribution, histological subtypes, and immunomorphological characteristics of all lymphomas with a primary GI presentation at a quaternary care hospital in southern India.

Methods

We conducted a retrospective study covering the period from 2018 to 2022 involving all cases of lymphomas with primary GI presentation. All the cases were categorized based on morphological and immunophenotypic criteria per the World Health Organization (WHO) 2022 classification of hematopoietic and lymphoid neoplasms. Data were analyzed by using the statistical software SPSS Statistics version 28.0 (IBM Corp., Armonk, NY).

Results

Out of 2,455 lymphoma cases diagnosed in the five-year study period, 156 presented with primary GI symptoms. Males constituted 113 (72.5%) cases and 43 (27.5%) patients were females. The stomach (n=81; 52%) was the most common site, followed by the small intestine (n=40; 27%), large intestine (n=33; 20%), and esophagus (n=2; 1%). Most lymphomas involving the GI tract were NHL (n=152; 97%), and the remaining included four (3%) cases of HL. Diffuse large B-cell lymphoma (DLBCL) was the most frequent (n=102; 65.3%) B-cell NHL. Eight cases of T-cell lymphomas were noted including rare entities.

Conclusions

This study is one of the largest of its kind involving lymphomas with primary GI presentation from India. The distribution and incidence of various entities in our study were comparable to various other studies from India and those from Western countries. GI lymphomas are heterogeneous, with distinct prognoses and treatment strategies. Hence, diagnosing them correctly would help in prognostication and providing prompt therapy.

## Introduction

Gastrointestinal (GI) tract lymphomas comprise 1-4% of all malignant neoplasms of the GI tract. Up to 40% of all extranodal non-Hodgkin lymphomas (NHLs) occur in the GI tract, making it the most common extranodal lymphoma site globally and constitutes approximately 10-15% of all NHL cases [1]. The disease's rarity and diverse clinical presentation preclude detection at an early stage when a cure is feasible [2]. The lymphomas' involvement in the GI tract can be primary or secondary as a part of systemic disease. Dawson et al. defined primary GI lymphomas as lymphomas that predominantly involve the GI tract with lymph node involvement confined to the drainage area of the primary tumor site, with no systemic involvement [3]. Studies have shown variations in the incidence of different histological subtypes and sites of involvement [4,5]. In this study, we aim to examine the demographic aspects related to lymphomas with primary GI presentation, classify GI lymphomas based on morphology and immunohistochemistry, and analyze the immunomorphological characteristics of GI lymphomas.

## Materials and methods

The study was conducted in the Department of Histopathology at a quaternary care hospital in Chennai, India. We employed a retrospective study design, covering a period of five years from 2018 to 2022. Inclusion criteria included all biopsies and resection cases of lymphomas involving the GI tract, including both in-house and referral cases. Extranodal lymphomas of the oral cavity, as well as pancreatic, hepatic, splenic, and other extraintestinal extranodal lymphomas, were excluded from the study. This study was approved by the Institutional Ethical Committee-Biomedical Research of Apollo Hospitals Greams Road Chennai (approval no: ASH-C-S-015/07-23).

The tissue was fixed in 10% neutral buffered formalin, processed, and paraffin-embedded. 4μ sections were cut and stained with hematoxylin and eosin (H&E). Immunohistochemistry (IHC) testing was performed with the VENTANA immunostaining platform using Optiview DAB with appropriate positive and negative controls. An appropriate panel of antibodies was used for each case as deemed necessary.

Data collection involved reviewing and classifying cases based on morphological and immunophenotypic criteria per the World Health Organization (WHO) 2022 classification of hematopoietic and lymphoid neoplasms. Genetic and molecular data were not included in the study. Data entry was performed using Microsoft Excel with proper coding. Clinical data was obtained from the hospital's electronic medical records.

Statistical analysis

Descriptive statistics were presented with mean ± standard deviation (SD) and frequency (percentage) for the continuous and categorical factors; also, the median was additionally reported for the continuous factors due to skewed values. No comparisons were performed in this study and hence p-value was not employed. Data were analyzed by using the statistical software SPSS Statistics version 28.0 (IBM Corp., Armonk, NY).

## Results

A total of 2,455 lymphoma cases were diagnosed in the five-year study period, out of which 156 (6.35%) were diagnosed in the GI tract specimens of patients who presented with primary GI symptoms. The gender distribution was as follows: 113 (72.5%) males and 43 (27.5%) females (male-to-female ratio: 2.62:1). The majority of the patients were in the older age groups, with a peak incidence in the sixth decade of life. The median age was 48 (range: 5-96 years), and there were nine pediatric cases. Of the 156 cases, 127 (82.5%) were received as endoscopy specimens, and 27 (17.5%) were resection cases. The most common site was the stomach (n=81; 52%), followed by the small intestine (n=40; 27%), large intestine (n=33; 20%), and esophagus (n=2; 1%). A complete history was only available in some cases, as many were referral cases from outside hospitals. There was one renal transplant recipient and one hepatitis B-positive patient. Of note, 152 cases were NHL (97%), and four (3%) cases were Hodgkin lymphoma (HL). B-cell lymphomas were the most common variant (n=144; 92%) (Table 1).

**Table 1:** Lymphomas with primary gastrointestinal presentation

Diagnosis	Esophagus, n=2 (1.28%), n (%)	Stomach, n=81 (52%), n (%)	Small intestine, n=40 (25.6%), n (%)	Large intestine, n=33 (21.1%), n (%)	Total (n=156), n (%)
						Diffuse large B-cell lymphoma	0	58 (37%)	23 (14.7%)	21 (13.4%)	102 (65.3%)
						High-grade B-cell lymphoma	1 (0.6%)	8 (5%)	4 (2.5%)	1 (0.6%)	14 (8.9%)
Burkitt lymphoma	1 (0.6%)	2 (1.28%)	0	1 (0.6%)	4 (2.5%)
Extranodal marginal zone lymphoma of mucosa-associated lymphoid tissue	0	4 (2.5%	3 (1.9%)	4 (2.5%)	11 (7%)
Mantle cell lymphoma	0	1 (0.6%)	3 (1.9%)	3 (1.9%)	7 (4.4%)
Small B-cell lymphoma, unclassified	0	1 (0.6%)	0	1 (0.6%)	2 (1.28%)
Follicular lymphoma	0	2 (1.28%)	2 (1.28%)	0	4 (2.5%)
Peripheral T-cell lymphoma, not otherwise specified	0	1 (0.6%)	1 (0.6%)	0	2 (1.28%)
Enteropathy-associated intestinal T-cell lymphoma	0	0	1 (0.6%)	0	1 (0.6%)
Anaplastic large cell lymphoma	0	0	1 (0.6%)	1 (0.6%)	2 (1.28%)
Monomorphic epitheliotropic T-cell lymphoma	0	0	1 (0.6%)	0	1 (0.6%)
Extranodal NK/T-cell lymphoma	0	2 (1.28%)	0	0	2 (1.28%)
Hodgkin lymphoma	0	2 (1.28%)	1 (0.6%)	1 (0.6%)	4 (2.5%)

**Figure 1:**
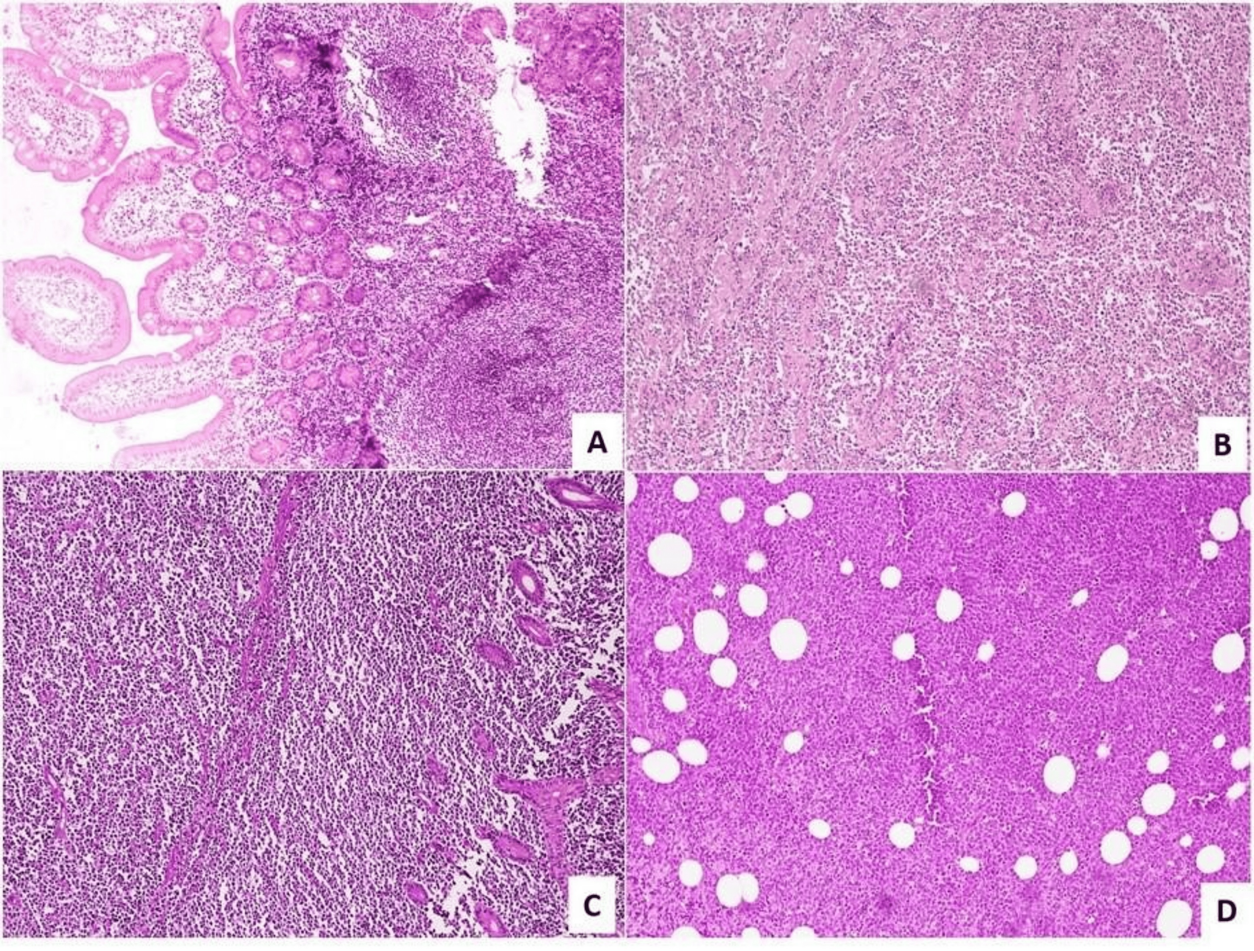
Immunohistochemistry analysis - 1 A: follicular lymphoma (H&E 100X); B: marginal zone lymphoma (H&E 400X); C: Burkitt lymphoma (H&E 400X); D: pleomorphic mantle cell lymphoma (H&E 400X)

**Figure 2:**
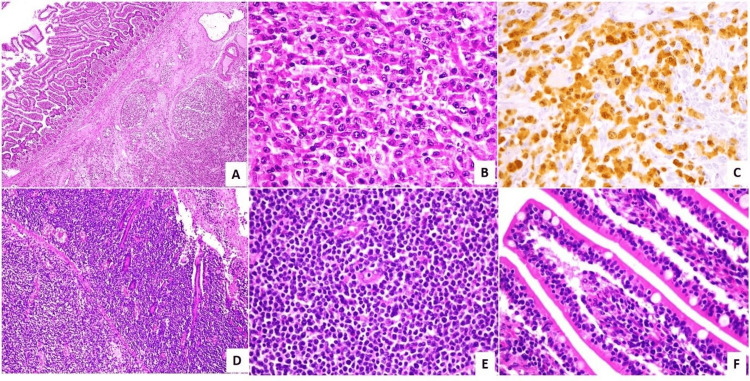
Immunohistochemistry analysis - 2 A and B: anaplastic large cell lymphoma (A - H&E 100X, B - H&E 400X); C: ALK-1 nuclear and cytoplasmic positivity (IHC 400X); D and E: monomorphic epitheliotropic intestinal T-cell lymphoma (D - H&E 100X), E - H&E 400X); F: increased intraepithelial lymphocytes (H&E 400X)

Immunohistochemistry analysis helped us to classify and subtype the various categories (Figures 1-2).

B-cell lymphomas in this study comprised extranodal marginal zone lymphoma of mucosa-associated lymphoid tissue (MALT lymphoma; n=11; 7%), mantle cell lymphoma (n=7; 4.4%), follicular lymphoma (n=4; 2.5%), diffuse large B-cell lymphoma (DLBCL; n=102; 65.3%), high-grade B-cell lymphoma (n=14; 8.9%), and Burkitt lymphoma (n=4; 2.5%); two cases were small B-cell NHL unclassified due to inadequate tissue for completing the workup. The cell of origin classification was done using the Hans algorithm. Among DLBCL cases, 55 were germinal center B-cell-like (GCB) lymphoma 47 were non-GCB; 23 cases were double expressors (C-MYC and BCL2-positive), and 13 cases were found to be triple expressors (C-MYC, BCL-2, and BCL-6-positive].

Helicobacter pylori (H. pylori) association was found in 10% (n=16) of cases. This included MALT (n=6) and DLBCL (n=10). Epstein-Barr virus (EBV)-encoding RNA in situ hybridization (EBER-ISH) was done in 72 cases, and 12 cases were variably positive. EBV was significantly positive in three cases and was labeled as EBV-positive DLBCL. One case of EBV-positive mucocutaneous ulcer (EBV-MCU) was identified in a 60-year-old male in the stomach with no obvious history of immunosuppression. Both the renal transplant recipient patient and hepatitis B-positive patient in our study were diagnosed with DLBCL. None of the cases with high-grade morphology expressed any primitive markers such as TdT, CD99, or CD34, thereby excluding lymphoblastic lymphoma. Most of them were GCB (eight cases) and the rest were non-GCB (six cases). All these cases had a high proliferation index with a mean of 80%. Among these cases, four were double expressors, and none were triple expressors. Molecular analysis was done in some cases, details of which are not included in this study.

T-cell lymphomas were infrequent and constituted only 5% (n=8) of cases. Two cases each of peripheral T-cell lymphoma not otherwise specified and extranodal NK/T-cell lymphomas and one case each of ALK-positive anaplastic large cell lymphoma, ALK-negative anaplastic large cell lymphoma, monomorphic epitheliotropic T-cell lymphoma, and enteropathy-associated intestinal T-cell lymphoma (EATL) were identified. No history of coeliac disease was documented in the EATL case. Four cases of classical HL involving the GI tract were included in our study. On further investigation, all the cases were found to be secondarily involving the GI tract. Treatment history and follow-up were not discussed in the study, as it was unavailable in most of the cases.

## Discussion

The GI tract is the most common site of extranodal NHL, accounting for as high as 40% of all extranodal NHL cases and approximately 10-15% of all NHL cases as per several studies from Western countries and Asia [1,2,6]; however, our study had a lower incidence (6.35%). Although primary GI tract lymphoma is rare, its incidence has been rising over the past 20 years. The stomach is the most common site of GI lymphoma, as reported by Koch et al., followed by small and large intestines, which aligns with our research [7].

In Middle Eastern countries, the incidence of intestinal lymphoma is high due to the high prevalence of immunoproliferative small intestinal disease (IPSID) [5]. In line with our study, a significant increase in incidence has been reported after the age of 40; the incidence peaks in the sixth decade of life [1,2,8,9]. Arora et al. and Malipatel et al. [9,10] have shown that primary gastric lymphoma is two to three times more common in men than women, which aligns with our study. Clinical presentation is variable, marked by nonspecific symptoms like abdominal pain, vomiting, constipation, diarrhea, and intestinal obstruction [7,10].

While the etiopathogenesis of GI lymphomas is not well understood, it has been determined that a variety of environmental, infectious, and genetic variables predispose individuals to lymphoma. Repetitive antigen stimulation by infection or inflammation has been proposed to lead to the development of GI lymphoma. Up to 90% of patients have H. pylori infection with MALT lymphoma, which was seen at a rate of 54% in our study [11,12]. HIV, EBV, hepatitis B virus, and human T-cell lymphotropic virus-1 are some of the other infections that have been linked to MALT lymphomas by various authors. Recent studies have illustrated the role of Campylobacter jejuni in the development of IPSID. Other conditions associated with gastric lymphoma development include inflammatory bowel diseases and chronic immunosuppression. Enteropathy-associated T-cell lymphoma is related to coeliac disease, which is more common in Western countries [12,13].

Determining the distribution pattern of various histological subtypes is critical because several studies have shown them to be independent prognostic factors [8,11]. Regardless of location, the majority of NHL cases affecting the GI tract are of the B-cell lineage, and most of these lymphomas are DLBCL and MALT lymphomas. Our study documented 62.8% of DLBCL cases among NHL patients, similar to the study conducted by Malipatel et al. [10]. Primary GI tract T-cell lymphomas are rare and constituted only 5% (n=8) in our study. Koch et al found T-cell NHL in 1.5% of all GI tract lymphomas in their German population [7]. Arora et al. conducted a study in southern India and documented 3.27% T-cell lymphomas among GI tract cases [9]. Primary HLs of the GI tract are rare, with only a handful of cases reported in the literature; these are often secondary to disseminated disease.

GI lymphomas, in general, have a variable prognosis depending on the histopathological, immunohistochemical, cytogenetical, biochemical, and clinical variables. The American Cancer Society provides survival statistics for different lymphomas and the International Prognostic Index (IPI) aids in the prognosis of each lymphoma based on specific parameters [12,13,14]. In GI tract cases, the site is also an independent variable for prognosis as complications due to obstruction and perforation vary in different sites [12].

Low-grade GI lymphomas such as MALT lymphomas and follicular lymphomas have an indolent course. Mantle cell lymphoma with a Ki67 >30% is associated with poor outcomes. Burkitt lymphoma should be addressed as an oncologic emergency due to the high proliferation index. Other high-grade B-cell lymphomas like DLBCL and T-cell lymphomas have a poor prognosis if treatment is delayed. The International T-cell Lymphoma Project has shown poor clinical outcomes in T-cell lymphomas in general [15,16]. Intestinal obstruction, perforation, paraneoplastic syndromes, transformation of indolent lymphomas to high-grade lymphomas, and adverse effects of chemotherapy and radiotherapy are the main complications of GI tract lymphomas.

Management strategies for lymphomas are tailor-made and potentially range from antibiotic therapy in the case of H. pylori-associated gastric MALT lymphoma to chemotherapy with or without radiation and, in rare cases, surgery. Duodenal follicular lymphoma cases receive conservative treatment with wait-and-watch monitoring [17,18]. Surgery is considered only in instances of obstruction, perforation, or when bleeding cannot be controlled endoscopically [18].

Limitations

This study has a few limitations, including the lack of follow-up, the absence of detailed clinical histories related to coeliac disease, and transplantation history, which hindered any potential insights into correlation.

## Conclusions

This study is one of the largest retrospective studies of lymphomas with primary GI presentation from India. The distribution and incidence of various entities in our study were comparable to other studies from India as well as those from Western countries. Our study demonstrated the pattern of distribution and various histological subtypes of GI lymphoma, including rare variants. GI lymphomas are heterogeneous, with differences in prognosis and different treatment modalities. Hence, diagnosing them correctly would help in treatment decisions and aid in prognosis.
